# 2-Bromo-1-(3-nitro­phen­yl)ethanone

**DOI:** 10.1107/S1600536810049585

**Published:** 2010-12-04

**Authors:** Jerry P. Jasinski, Ray J. Butcher, A. S. Praveen, H. S. Yathirajan, B. Narayana

**Affiliations:** a Department of Chemistry, Keene State College, 229 Main Street, Keene, NH 03435-2001, USA; b Department of Chemistry, Howard University, 525 College Street NW, Washington, DC 20059, USA; c Department of Studies in Chemistry, University of Mysore, Manasagangotri, Mysore 570 006, India; d Department of Studies in Chemistry, Mangalore University, Mangalagangotri 574 199, India

## Abstract

In the title compound, C_8_H_6_BrNO_3_, there are two mol­ecules, *A* and *B*, in the asymmetric unit. The nitro and ethanone groups lie close to the plane of the benzene ring and the bromine atom is twisted slightly: the dihedral angles between the mean planes of the nitro and ethanone groups and the benzene ring are 4.6 (4) (*A*) and 2.8 (3) (*B*), and 0.8 (8) (*A*) and 5.5 (8)° (*B*), respectively. An extensive array of weak C—H⋯O hydrogen bonds, π–π ring stacking [centroid–centroid distances = 3.710 (5) and 3.677 (5) Å] and short non-hydrogen Br⋯O and O⋯Br inter­molecular inter­actions [3.16 (6)and 3.06 (8) Å] contribute to the crystal stability, forming a supermolecular three-dimensional network structure along 110. These inter­actions give rise to a variety of cyclic graph-set motifs and form inter­connected sheets in the three-dimensional structure.

## Related literature

For the use of α-haloketones in the synthesis of pharmaceuticals, see: Erian *et al.* (2003 ▶). For related structures, see: Gupta & Prasad (1971 ▶); Sim (1986 ▶); Sutherland & Hoy (1968 ▶, 1969 ▶); Sutherland *et al.* (1974 ▶); Yathirajan *et al.* (2007 ▶); Young *et al.* (1968 ▶). For cyclic graph-set motifs, see: Etter (1990 ▶). For reference bond-length data, see: Allen *et al.* (1987 ▶).
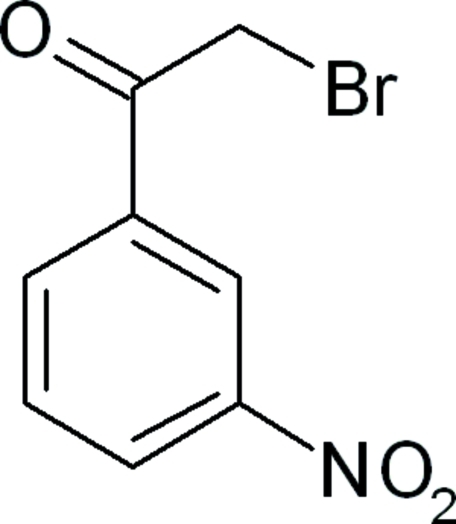


## Experimental

### 

#### Crystal data

C_8_H_6_BrNO_3_
*M* *_r_* = 244.05
Triclinic, 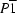

*a* = 8.8259 (7) Å
*b* = 8.8651 (8) Å
*c* = 11.6775 (8) Å
α = 74.691 (7)°
β = 75.174 (7)°
γ = 78.681 (7)°
*V* = 843.76 (12) Å^3^
*Z* = 4
Cu *K*α radiation
μ = 6.45 mm^−1^
*T* = 123 K
0.75 × 0.62 × 0.19 mm

#### Data collection

Oxford Diffraction Xcalibur Ruby Gemini diffractometer
Absorption correction: analytical (*CrysAlis RED*; Oxford Diffraction, 2007 ▶) *T*_min_ = 0.066, *T*_max_ = 0.389
4708 measured reflections
3215 independent reflections
3023 reflections with *I* > 2σ(*I*)
*R*_int_ = 0.053

#### Refinement

*R*[*F* ^2^ > 2σ(*F* ^2^)] = 0.090
*wR*(*F* ^2^) = 0.248
*S* = 1.12
3215 reflections
235 parameters
H-atom parameters constrained
Δρ_max_ = 2.39 e Å^−3^
Δρ_min_ = −1.83 e Å^−3^

### 

Data collection: *CrysAlis PRO* (Oxford Diffraction, 2007 ▶); cell refinement: *CrysAlis PRO*; data reduction: *CrysAlis RED* (Oxford Diffraction, 2007 ▶); program(s) used to solve structure: *SHELXS97* (Sheldrick, 2008 ▶); program(s) used to refine structure: *SHELXL97* (Sheldrick, 2008 ▶)); molecular graphics: *SHELXTL* (Sheldrick, 2008 ▶); software used to prepare material for publication: *SHELXTL*.

## Supplementary Material

Crystal structure: contains datablocks global, I. DOI: 10.1107/S1600536810049585/sj5067sup1.cif

Structure factors: contains datablocks I. DOI: 10.1107/S1600536810049585/sj5067Isup2.hkl

Additional supplementary materials:  crystallographic information; 3D view; checkCIF report

## Figures and Tables

**Table 1:** Hydrogen-bond geometry (Å, °)

*D*—H⋯*A*	*D*—H	H⋯*A*	*D*⋯*A*	*D*—H⋯*A*
C4*A*—H4*AA*⋯O1*B*^i^	0.95	2.49	3.314 (10)	145
C5*A*—H5*AA*⋯Br2^ii^	0.95	3.04	3.849 (8)	144
C5*A*—H5*AA*⋯O2*B*^i^	0.95	2.55	3.409 (11)	150
C6*A*—H6*AA*⋯O3*B*^ii^	0.95	2.38	3.320 (10)	171
C4*B*—H4*BA*⋯O1*A*^iii^	0.95	2.56	3.420 (9)	150
C6*B*—H6*BA*⋯O3*A*	0.95	2.35	3.278 (10)	165

Symmetry codes: (i) 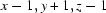; (ii) 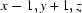; (iii) 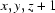.

**Fig. 1.**
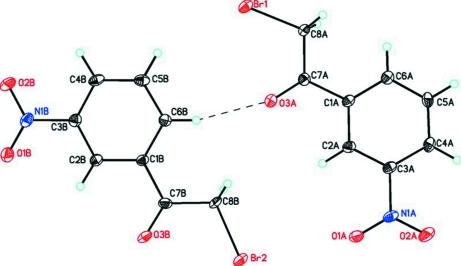
Molecular structure of the title compound showing the atom labeling scheme and 50% probability displacement ellipsoids. Dashed lines indicate weak C—H···O intermolecular hydrogen bonds between two molecules in the asymmetric unit.

**Fig. 2.**
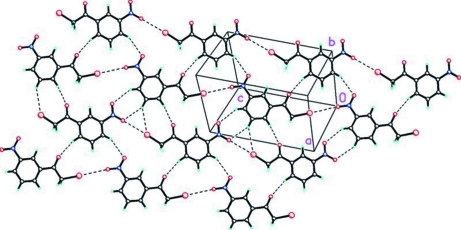
Packing diagram of the title compound viewed down the a axis. Dashed lines indicate weak C—H···O and C—H···Br hydrogen bonds and short non-hydrogen, Br···O and O···Br, intermolecular interactions creating a 3-D supramolecular structure along 110.

**Fig. 3.**
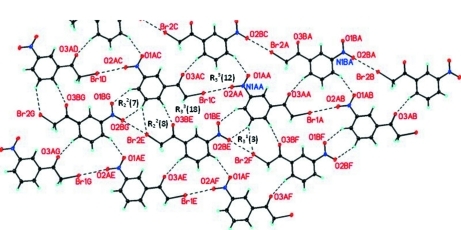
A planar sheet of C8H6BrNO3 molecules connected by weak C—H···O and C—H···Br hydrogen bonds and short non-hydrogen, Br···O and O···Br intermolecular interactions. These patterns are shown by cyclic graph-set motif analysis (R31(3), R22(7), R22(8), R33(12), R33(18)) in an extended 2-dimensional array.

## supplementary crystallographic information

### Comment

α-Haloketones have been attracting increasing attention in view of their high reactivity as building blocks for the preparation of compounds of various classes due to their selective transformations with different reagents.  The α-haloketones can be particularly promising synthons in combinatorial synthesis of functionalized carbo- and heterocyclic compounds used in the design of novel highly effective pharmaceuticals with a broad spectrum of bioresponses (Erian *et al.*, 2003). Crystal structures of some acetyl biphenyl derivatives viz., 4-acetyl-2'-fluorobiphenyl (Young *et al.*, 1968), 4-acetyl-2'-chlorobiphenyl (Sutherland & Hoy, 1968), 4-acetyl-3'-bromobiphenyl (Sutherland & Hoy, 1969), 4-acetyl-2'-nitrobiphenyl (Sutherland *et al.*, 1974), α-bromoacetophenone (Gupta & Prasad, 1971), 2-Bromo-4'-phenylacetophenone (Sim, 1986 ) and methyl 4-(bromomethyl)benzoate (Yathirajan *et al.*2007) have been reported.  In view of the importance of the α-haloketones, the title compound, (I), has been prepared and its crystal structure is reported.

In the title compound, C_8_H_6_BrNO_3_, two molecules crystallize in the asymmetric unit (Fig. 2).  The nitro and ethanone groups are planar with the benzene ring and the bromine atom is twisted slightly (Torsion angles C1A/C7A/C8A/Br1 = -177.5 (5)° and C1B/C7B/C8B/Br2 = 168.6 (5)°. Bond distances and angles are in normal ranges (Allen *et al.*, 1987). An extensive array of weak C—H···O and C—H···Br  hydrogen bonds (Table 1), π–π ring stacking (Table 2) and short non-hydrogen, Br···O and O···Br, intermolecular interactions (Table 3) contribute to crystal stability forming a supermolecular 3-dimensional network structure along 110 (Fig. 3). These interactions give rise to a variety of cyclic graph-set motifs (R_3_^1^(3), R_2_^2^(7), R_2_^2^(8), R_3_^3^(12), R_3_^3^(18)), Fig. 3, (Etter, 1990) and form interconnected sheets in the three-dimensional structure.

### Experimental

To a stirred solution of 1-(3-nitrophenyl)ethanone (1 g, 6.05 mmol) in chloroform (10 ml), bromine (0.97 g, 6.05 mmol) was added at 0–5°C (Fig. 1). The reaction mixture was stirred at room temperature for 2 h, poured into ice cold water and layers were separated. The organic layer was washed with water (1 *x* 10 ml), 10% aq.sodium bicarbonate solution (1 *x* 10 ml) and brine (1 *x* 10 ml), dried over Na_2_SO_4_ and concentrated under reduced pressure. The crude product was purified by column chromatography in silca gel (230–400 mesh) using 0–10% petroleum ether and ethyl acetate as the elutant. Single crystals were grown from THF by the slow evaporation method with a yield of 96% (m.p.365–367 K).

### Refinement

All of the H atoms were placed in their calculated positions and refined using the riding model with Atom—H lengths of 0.95Å (CH) or 0.99Å (CH_2_). Isotropic displacement parameters for these atoms were set to 1.19–1.22 (CH) or 1.18–1.20 (CH_2_) times *U*_eq_ of the parent atom.

### Crystal data

**Table:**

C_8_H_6_BrNO_3_	*Z* = 4
*M**_r_* = 244.05	*F*(000) = 480
Triclinic, *P*1	*D*_x_ = 1.921 Mg m^−^^3^
Hall symbol: -P 1	Cu *K*α radiation, λ = 1.54178 Å
*a* = 8.8259 (7) Å	Cell parameters from 4487 reflections
*b* = 8.8651 (8) Å	θ = 5.2–74.4°
*c* = 11.6775 (8) Å	µ = 6.45 mm^−^^1^
α = 74.691 (7)°	*T* = 123 K
β = 75.174 (7)°	Plate, colorless
γ = 78.681 (7)°	0.75 × 0.62 × 0.19 mm
*V* = 843.76 (12) Å^3^	

### Data collection

**Table:**

Oxford Diffraction Xcalibur Ruby Gemini diffractometer	3215 independent reflections
Radiation source: Enhance (Cu) X-ray Source	3023 reflections with *I* > 2σ(*I*)
graphite	*R*_int_ = 0.053
Detector resolution: 10.5081 pixels mm^-1^	θ_max_ = 74.5°, θ_min_ = 5.2°
ω scans	*h* = −10→10
Absorption correction: analytical (*CrysAlis RED*; Oxford Diffraction, 2007)	*k* = −10→11
*T*_min_ = 0.066, *T*_max_ = 0.389	*l* = −10→14
4708 measured reflections	

### Refinement

**Table:**

Refinement on *F*^2^	Primary atom site location: structure-invariant direct methods
Least-squares matrix: full	Secondary atom site location: difference Fourier map
*R*[*F*^2^ > 2σ(*F*^2^)] = 0.090	Hydrogen site location: inferred from neighbouring sites
*wR*(*F*^2^) = 0.248	H-atom parameters constrained
*S* = 1.12	*w* = 1/[σ^2^(*F*_o_^2^) + (0.1498*P*)^2^ + 8.1184*P*] where *P* = (*F*_o_^2^ + 2*F*_c_^2^)/3
3215 reflections	(Δ/σ)_max_ < 0.001
235 parameters	Δρ_max_ = 2.39 e Å^−^^3^
0 restraints	Δρ_min_ = −1.83 e Å^−^^3^

### Special details

**Table:**

Geometry. All e.s.d.'s (except the e.s.d. in the dihedral angle between two l.s. planes) are estimated using the full covariance matrix. The cell e.s.d.'s are taken into account individually in the estimation of e.s.d.'s in distances, angles and torsion angles; correlations between e.s.d.'s in cell parameters are only used when they are defined by crystal symmetry. An approximate (isotropic) treatment of cell e.s.d.'s is used for estimating e.s.d.'s involving l.s. planes.
Refinement. Refinement of *F*^2^ against ALL reflections. The weighted *R*-factor *wR* and goodness of fit *S* are based on *F*^2^, conventional *R*-factors *R* are based on *F*, with *F* set to zero for negative *F*^2^. The threshold expression of *F*^2^ > σ(*F*^2^) is used only for calculating *R*-factors(gt) *etc*. and is not relevant to the choice of reflections for refinement. *R*-factors based on *F*^2^ are statistically about twice as large as those based on *F*, and *R*- factors based on ALL data will be even larger.

### Fractional atomic coordinates and isotropic or equivalent isotropic displacement parameters (Å^2^)

**Table:**

	*x*	*y*	*z*	*U*_iso_*/*U*_eq_	
Br1	0.22668 (10)	0.51160 (10)	0.93394 (7)	0.0303 (3)	
Br2	0.72920 (10)	−0.00313 (10)	0.49047 (7)	0.0301 (3)	
O1A	0.4973 (7)	0.2908 (7)	0.3052 (5)	0.0323 (13)	
O2A	0.3515 (9)	0.4156 (10)	0.1781 (6)	0.0471 (17)	
O3A	0.3683 (8)	0.3750 (8)	0.7146 (5)	0.0341 (14)	
O1B	0.9660 (7)	−0.2309 (7)	1.1190 (6)	0.0343 (13)	
O2B	0.8390 (10)	−0.0778 (11)	1.2376 (7)	0.056 (2)	
O3B	0.8567 (9)	−0.1476 (9)	0.7169 (6)	0.0470 (18)	
N1A	0.3867 (8)	0.3869 (8)	0.2772 (6)	0.0279 (14)	
N1B	0.8677 (8)	−0.1185 (9)	1.1406 (6)	0.0300 (15)	
C1A	0.2294 (9)	0.5263 (9)	0.5648 (7)	0.0213 (14)	
C2A	0.3214 (9)	0.4390 (9)	0.4807 (7)	0.0230 (15)	
H2AA	0.4027	0.3562	0.5020	0.028*	
C3A	0.2895 (9)	0.4778 (9)	0.3663 (7)	0.0234 (15)	
C4A	0.1708 (9)	0.5973 (9)	0.3319 (7)	0.0260 (16)	
H4AA	0.1522	0.6209	0.2520	0.031*	
C5A	0.0816 (10)	0.6799 (9)	0.4154 (8)	0.0268 (16)	
H5AA	−0.0004	0.7614	0.3936	0.032*	
C6A	0.1098 (9)	0.6459 (9)	0.5319 (7)	0.0250 (15)	
H6AA	0.0474	0.7044	0.5892	0.030*	
C7A	0.2640 (9)	0.4810 (9)	0.6892 (7)	0.0231 (15)	
C8A	0.1639 (10)	0.5758 (9)	0.7794 (7)	0.0262 (15)	
H8AA	0.0515	0.5626	0.7923	0.031*	
H8AB	0.1733	0.6891	0.7451	0.031*	
C1B	0.7227 (9)	0.0124 (9)	0.8533 (7)	0.0210 (14)	
C2B	0.8084 (9)	−0.0752 (9)	0.9417 (7)	0.0221 (14)	
H2BA	0.8829	−0.1653	0.9282	0.027*	
C3B	0.7801 (9)	−0.0256 (9)	1.0474 (7)	0.0239 (15)	
C4B	0.6745 (9)	0.1066 (9)	1.0720 (7)	0.0258 (16)	
H4BA	0.6607	0.1374	1.1464	0.031*	
C5B	0.5909 (10)	0.1913 (9)	0.9858 (8)	0.0272 (16)	
H5BA	0.5175	0.2816	1.0005	0.033*	
C6B	0.6136 (9)	0.1452 (8)	0.8766 (7)	0.0224 (15)	
H6BA	0.5548	0.2039	0.8176	0.027*	
C7B	0.7549 (9)	−0.0401 (9)	0.7370 (7)	0.0252 (15)	
C8B	0.6500 (9)	0.0468 (10)	0.6461 (7)	0.0249 (15)	
H8BA	0.6431	0.1621	0.6367	0.030*	
H8BB	0.5418	0.0182	0.6791	0.030*	

### Atomic displacement parameters (Å^2^)

**Table:**

	*U*^11^	*U*^22^	*U*^33^	*U*^12^	*U*^13^	*U*^23^
Br1	0.0385 (5)	0.0349 (5)	0.0186 (5)	−0.0091 (4)	−0.0038 (3)	−0.0072 (3)
Br2	0.0404 (6)	0.0338 (5)	0.0163 (5)	−0.0076 (4)	−0.0030 (3)	−0.0075 (3)
O1A	0.041 (3)	0.029 (3)	0.022 (3)	0.000 (3)	0.000 (2)	−0.008 (2)
O2A	0.055 (4)	0.064 (5)	0.027 (3)	0.004 (3)	−0.015 (3)	−0.022 (3)
O3A	0.040 (3)	0.037 (3)	0.022 (3)	0.007 (3)	−0.010 (2)	−0.007 (2)
O1B	0.041 (3)	0.032 (3)	0.030 (3)	0.002 (3)	−0.015 (3)	−0.006 (2)
O2B	0.073 (5)	0.071 (5)	0.024 (3)	0.021 (4)	−0.019 (3)	−0.025 (3)
O3B	0.063 (4)	0.055 (4)	0.020 (3)	0.018 (4)	−0.014 (3)	−0.019 (3)
N1A	0.032 (3)	0.031 (3)	0.019 (3)	−0.010 (3)	0.005 (3)	−0.008 (3)
N1B	0.034 (4)	0.038 (4)	0.018 (3)	−0.008 (3)	−0.006 (3)	−0.003 (3)
C1A	0.023 (3)	0.024 (3)	0.018 (4)	−0.009 (3)	−0.002 (3)	−0.004 (3)
C2A	0.025 (4)	0.023 (4)	0.018 (4)	−0.007 (3)	0.002 (3)	−0.003 (3)
C3A	0.025 (4)	0.026 (4)	0.018 (4)	−0.011 (3)	0.002 (3)	−0.005 (3)
C4A	0.031 (4)	0.024 (4)	0.020 (4)	−0.012 (3)	−0.004 (3)	0.003 (3)
C5A	0.031 (4)	0.021 (4)	0.027 (4)	−0.006 (3)	−0.010 (3)	0.002 (3)
C6A	0.027 (4)	0.025 (4)	0.023 (4)	−0.008 (3)	−0.004 (3)	−0.003 (3)
C7A	0.028 (4)	0.019 (3)	0.021 (4)	−0.007 (3)	−0.005 (3)	−0.002 (3)
C8A	0.035 (4)	0.026 (4)	0.018 (4)	0.001 (3)	−0.008 (3)	−0.006 (3)
C1B	0.025 (3)	0.021 (3)	0.016 (3)	−0.004 (3)	−0.002 (3)	−0.004 (3)
C2B	0.023 (3)	0.024 (4)	0.017 (3)	−0.006 (3)	0.000 (3)	−0.003 (3)
C3B	0.025 (4)	0.027 (4)	0.017 (4)	−0.006 (3)	−0.002 (3)	−0.001 (3)
C4B	0.033 (4)	0.028 (4)	0.015 (3)	−0.012 (3)	0.005 (3)	−0.007 (3)
C5B	0.028 (4)	0.025 (4)	0.025 (4)	−0.006 (3)	0.004 (3)	−0.008 (3)
C6B	0.027 (4)	0.018 (3)	0.018 (3)	−0.003 (3)	−0.001 (3)	0.001 (3)
C7B	0.025 (4)	0.026 (4)	0.022 (4)	−0.001 (3)	0.001 (3)	−0.008 (3)
C8B	0.031 (4)	0.033 (4)	0.014 (3)	−0.006 (3)	−0.005 (3)	−0.009 (3)

### Geometric parameters (Å, °)

**Table:**

Br1—C8A	1.932 (8)	C5A—H5AA	0.9500
Br2—C8B	1.908 (7)	C6A—H6AA	0.9500
O1A—N1A	1.215 (9)	C7A—C8A	1.515 (11)
O2A—N1A	1.224 (10)	C8A—H8AA	0.9900
O3A—C7A	1.213 (10)	C8A—H8AB	0.9900
O1B—N1B	1.221 (10)	C1B—C6B	1.406 (10)
O2B—N1B	1.229 (10)	C1B—C2B	1.410 (11)
O3B—C7B	1.202 (10)	C1B—C7B	1.492 (11)
N1A—C3A	1.477 (10)	C2B—C3B	1.366 (11)
N1B—C3B	1.472 (10)	C2B—H2BA	0.9500
C1A—C6A	1.392 (11)	C3B—C4B	1.392 (11)
C1A—C2A	1.403 (11)	C4B—C5B	1.373 (12)
C1A—C7A	1.496 (11)	C4B—H4BA	0.9500
C2A—C3A	1.377 (11)	C5B—C6B	1.394 (12)
C2A—H2AA	0.9500	C5B—H5BA	0.9500
C3A—C4A	1.392 (12)	C6B—H6BA	0.9500
C4A—C5A	1.365 (12)	C7B—C8B	1.539 (11)
C4A—H4AA	0.9500	C8B—H8BA	0.9900
C5A—C6A	1.390 (12)	C8B—H8BB	0.9900
			
O1A—N1A—O2A	124.0 (7)	C7A—C8A—H8AB	109.2
O1A—N1A—C3A	118.7 (7)	Br1—C8A—H8AB	109.2
O2A—N1A—C3A	117.3 (7)	H8AA—C8A—H8AB	107.9
O1B—N1B—O2B	122.5 (7)	C6B—C1B—C2B	119.5 (7)
O1B—N1B—C3B	119.5 (7)	C6B—C1B—C7B	122.9 (7)
O2B—N1B—C3B	118.0 (7)	C2B—C1B—C7B	117.6 (7)
C6A—C1A—C2A	120.2 (7)	C3B—C2B—C1B	117.4 (7)
C6A—C1A—C7A	123.0 (7)	C3B—C2B—H2BA	121.3
C2A—C1A—C7A	116.8 (7)	C1B—C2B—H2BA	121.3
C3A—C2A—C1A	117.5 (7)	C2B—C3B—C4B	124.2 (8)
C3A—C2A—H2AA	121.3	C2B—C3B—N1B	117.5 (7)
C1A—C2A—H2AA	121.3	C4B—C3B—N1B	118.3 (7)
C2A—C3A—C4A	123.0 (7)	C5B—C4B—C3B	118.2 (7)
C2A—C3A—N1A	117.7 (7)	C5B—C4B—H4BA	120.9
C4A—C3A—N1A	119.3 (7)	C3B—C4B—H4BA	120.9
C5A—C4A—C3A	118.6 (8)	C4B—C5B—C6B	120.2 (7)
C5A—C4A—H4AA	120.7	C4B—C5B—H5BA	119.9
C3A—C4A—H4AA	120.7	C6B—C5B—H5BA	119.9
C4A—C5A—C6A	120.6 (8)	C5B—C6B—C1B	120.5 (7)
C4A—C5A—H5AA	119.7	C5B—C6B—H6BA	119.7
C6A—C5A—H5AA	119.7	C1B—C6B—H6BA	119.7
C5A—C6A—C1A	120.1 (8)	O3B—C7B—C1B	121.0 (7)
C5A—C6A—H6AA	120.0	O3B—C7B—C8B	121.9 (7)
C1A—C6A—H6AA	120.0	C1B—C7B—C8B	117.1 (6)
O3A—C7A—C1A	121.0 (7)	C7B—C8B—Br2	112.4 (5)
O3A—C7A—C8A	122.6 (7)	C7B—C8B—H8BA	109.1
C1A—C7A—C8A	116.4 (7)	Br2—C8B—H8BA	109.1
C7A—C8A—Br1	112.2 (5)	C7B—C8B—H8BB	109.1
C7A—C8A—H8AA	109.2	Br2—C8B—H8BB	109.1
Br1—C8A—H8AA	109.2	H8BA—C8B—H8BB	107.8
			
C6A—C1A—C2A—C3A	0.7 (11)	C6B—C1B—C2B—C3B	0.1 (11)
C7A—C1A—C2A—C3A	179.1 (6)	C7B—C1B—C2B—C3B	179.2 (7)
C1A—C2A—C3A—C4A	−0.5 (11)	C1B—C2B—C3B—C4B	−1.1 (11)
C1A—C2A—C3A—N1A	179.7 (6)	C1B—C2B—C3B—N1B	179.1 (6)
O1A—N1A—C3A—C2A	−5.5 (10)	O1B—N1B—C3B—C2B	3.0 (11)
O2A—N1A—C3A—C2A	175.7 (7)	O2B—N1B—C3B—C2B	−177.6 (8)
O1A—N1A—C3A—C4A	174.7 (7)	O1B—N1B—C3B—C4B	−176.8 (7)
O2A—N1A—C3A—C4A	−4.1 (11)	O2B—N1B—C3B—C4B	2.6 (11)
C2A—C3A—C4A—C5A	−0.1 (11)	C2B—C3B—C4B—C5B	1.3 (12)
N1A—C3A—C4A—C5A	179.8 (7)	N1B—C3B—C4B—C5B	−178.9 (7)
C3A—C4A—C5A—C6A	0.5 (11)	C3B—C4B—C5B—C6B	−0.5 (11)
C4A—C5A—C6A—C1A	−0.3 (12)	C4B—C5B—C6B—C1B	−0.4 (12)
C2A—C1A—C6A—C5A	−0.3 (11)	C2B—C1B—C6B—C5B	0.6 (11)
C7A—C1A—C6A—C5A	−178.6 (7)	C7B—C1B—C6B—C5B	−178.4 (7)
C6A—C1A—C7A—O3A	179.3 (7)	C6B—C1B—C7B—O3B	174.9 (8)
C2A—C1A—C7A—O3A	0.9 (11)	C2B—C1B—C7B—O3B	−4.2 (12)
C6A—C1A—C7A—C8A	−1.5 (11)	C6B—C1B—C7B—C8B	−6.5 (11)
C2A—C1A—C7A—C8A	−179.9 (7)	C2B—C1B—C7B—C8B	174.5 (6)
O3A—C7A—C8A—Br1	1.7 (10)	O3B—C7B—C8B—Br2	−12.8 (10)
C1A—C7A—C8A—Br1	−177.5 (5)	C1B—C7B—C8B—Br2	168.6 (5)

### Hydrogen-bond geometry (Å, °)

**Table:**

*D*—H···*A*	*D*—H	H···*A*	*D*···*A*	*D*—H···*A*
C4A—H4AA···O1B^i^	0.95	2.49	3.314 (10)	145.
C5A—H5AA···Br2^ii^	0.95	3.04	3.849 (8)	144.
C5A—H5AA···O2B^i^	0.95	2.55	3.409 (11)	150.
C6A—H6AA···O3B^ii^	0.95	2.38	3.320 (10)	171.
C4B—H4BA···O1A^iii^	0.95	2.56	3.420 (9)	150.
C6B—H6BA···O3A	0.95	2.35	3.278 (10)	165.

Symmetry codes: (i) *x*−1, *y*+1, *z*−1; (ii) *x*−1, *y*+1, *z*; (iii) *x*, *y*, *z*+1.

### Table 2 Cg···Cg π stacking interactions (Å)

Cg1 and Cg2 are the centroids of rings C1A–C6A and Cg1B–Cg6B

**Table:**

*Cg*I···*Cg*J	Cg···Cg	CgI Perp	CgJ Perp	Slippage
Cg1···Cg1^i^	3.710 (5)	-3.357 (3)	-3.357 (3)	1.58 (2)
Cg2···Cg2^ii^	3.677 (5)	-3.418 (3)	-3.418 (3)	1.35 (5)

Symmetry codes: (i) -x, 1-y, 1-z; (ii) 1-x, -y, 2-z.

### Table 3 Short non-hydrogen intermolecular interactions (Å).

**Table:**

Atom I···Atom J	d(I–J)	Del
Br1^i^···O2A^ii^	3.16 (6)	-0.20
O2A^i^···Br1^iii^	3.16 (6)	-0.20
Br2^i^···O2B^iii^	3.06 (8)	-0.30
O2B^i^···Br2^ii^	3.06 (8)	-0.30

Symmetry codes: (i) x, y, z; (ii) x, y, 1+z; (iii) x, y, -1+z.

## References

[bb1] Allen, F. H., Kennard, O., Watson, D. G., Brammer, L., Orpen, A. G. & Taylor, R. (1987). *J. Chem. Soc. Perkin Trans. 2*, pp. S1–19.

[bb2] Erian, A. W., Sherif, S. M. & Gaber, H. M. (2003). *Molecules*, **8**, 793–865.

[bb3] Etter, M. C. (1990). *Acc. Chem. Res.* **23**, 120–126.

[bb4] Gupta, M. P. & Prasad, S. M. (1971). *Acta Cryst.* B**27**, 1649–1653.

[bb5] Oxford Diffraction (2007). *CrysAlis PRO* and *CrysAlis RED* Oxford Diffraction Ltd, Abingdon, England.

[bb6] Sheldrick, G. M. (2008). *Acta Cryst.* A**64**, 112–122. 10.1107/S0108767307043930 18156677

[bb7] Sim, G. A. (1986). *Acta Cryst.* C**42**, 1411–1413.

[bb8] Sutherland, H. H., Hogg, J. H. C. & Williams, D. J. (1974). *Acta Cryst.* B**30**, 1562–1565.

[bb9] Sutherland, H. H. & Hoy, T. G. (1968). *Acta Cryst.* B**24**, 1207–1213.

[bb10] Sutherland, H. H. & Hoy, T. G. (1969). *Acta Cryst.* B**25**, 2385–2391.

[bb11] Yathirajan, H. S., Bindya, S., Sarojini, B. K., Narayana, B. & Bolte, M. (2007). *Acta Cryst.* E**63**, o1334–o1335.

[bb12] Young, D. W., Tollin, P. & Sutherland, H. H. (1968). *Acta Cryst.* B**24**, 161–167.

